# Malnutrition at Admission Predicts In-Hospital Falls in Hospitalized Older Adults

**DOI:** 10.3390/nu12020541

**Published:** 2020-02-20

**Authors:** Yuria Ishida, Keisuke Maeda, Tomoyuki Nonogaki, Akio Shimizu, Yosuke Yamanaka, Remi Matsuyama, Ryoko Kato, Naoharu Mori

**Affiliations:** 1 Department of Nutrition, Aichi Medical University Hospital, Nagakute 480-1195, Japan; okuda.yuria.785@mail.aichi-med-u.ac.jp; 2 Department of Palliative and Supportive Medicine, Graduate School of Medicine, Aichi Medical University, Nagakute 480-1195, Japan; nmori@aichi-med-u.ac.jp; 3 Nutritional Therapy Support Center, Aichi Medical University Hospital, Nagakute 480-1195, Japan; 4 Department of Pharmacy, Aichi Medical University Hospital, Nagakute, Aichi 480-1195, Japan; nonogaki.tomoyuki.562@mail.aichi-med-u.ac.jp (T.N.); inuduka.ryouko.907@mail.aichi-med-u.ac.jp (R.K.); 5 Department of Nutrition, Hamamatsu City Rehabilitation Hospital, Hamamatsu 433-8511, Japan; a.shimizu.diet@gmail.com; 6 Department of Oral and Maxillofacial Surgery, Graduate School of Medicine, Aichi Medical University, Nagakute 480-1195, Japan; yamanaka.yousuke.694@mail.aichi-med-u.ac.jp (Y.Y.); matsuyama.remi.721@mail.aichi-med-u.ac.jp (R.M.)

**Keywords:** undernutrition, fall, hospitalization, older adult

## Abstract

Malnutrition leads to poor prognoses, including a predisposition to falls. Few studies have investigated the relationship between malnutrition and falls during hospitalization. This study aimed to determine malnutrition’s association with falls during hospitalization. A retrospective observational study was conducted. Patients aged ≥65 years that were admitted to and discharged from a university hospital between April 2018 and March 2019 were examined. Patients with independent basic activities of daily living were included. Diagnosis of malnutrition was based on the European Society for Clinical Nutrition and Metabolism (ESPEN) criteria at admission. Disease information such as the Charlson Comorbidity Index (CCI) and reasons for hospitalization were reviewed. Kaplan–Meier curve and multivariate Cox regression analyses were performed. Data from 6081 patients (mean age: 74.4 ± 6.1 years; males: 58.1%) were analyzed. The mean CCI was 2.3 ± 2.8 points. Malnutrition was detected in 668 (11.0%) and falls occurred in 55 (0.9%) patients. Malnourished patients experienced a higher fall rate than those without malnutrition (2.4% vs. 0.7%, log-rank test *p* < 0.001). In multivariate analysis, malnutrition had the highest hazard ratio for falls among covariates (hazard ratio 2.78, 95% confidence interval 1.51–5.00, *p* = 0.001). In conclusion, malnutrition at the time of admission to hospital predicts in-hospital falls.

## 1. Introduction

Falls are quite common in older adults, with about 30% of people aged ≥65 years falling every year [1]. If a community-dwelling elderly person falls, they are likely to fall again [1] or be hospitalized [2]. In addition, it is reported that falling is the most common adverse event requiring acute care tertiary hospitalization, with a profound adverse effect on patient outcomes, quality of life, and increased medical costs [3]. Risk factors for falls are known to include being elderly, impaired balance and gait, polypharmacy [4], and a high prevalence of comorbidities [5]. Decreased skeletal muscle mass and strength with the progression of age affects the balance function [6,7]. In addition, older, hospitalized patients spend most of their time lying in bed, despite an ability to walk independently [8], and low activity leads to further loss of muscle mass and strength. Furthermore, older patients admitted to acute care hospitals have many comorbidities and may be taking multiple drugs [9]. Therefore, older individuals hospitalized in acute settings are at risk of falling.

Malnutrition may result in an extended hospital stay, increased frequency of re-admission, increased mortality [10], and failure to return home [11]. Malnutrition is present in approximately 25% of hospitalized older adult patients in acute care [12]. Aging individuals are susceptible to malnutrition [12]. In addition, patients admitted to acute care hospitals may develop malnutrition due to inflammation caused by acute or chronic illness [13], decreased activity [8], or decreased nutrition intake [14]. Malnutrition often results in low skeletal muscle mass and low muscle strength, which increases the risk of falling [15,16,17]. In turn, the risk of falling may be related to adverse effects on patient outcomes, quality of life, and increased medical costs. However, not much is known about the relationship between malnutrition and falls during hospitalization. The purpose of this study was to investigate whether malnutrition is associated with in-hospital falls in an acute care hospital.

## 2. Materials and Methods

### 2.1. Study Design and Participants

A retrospective observational study was conducted. Older adult patients aged ≥65 years, admitted to and discharged from an acute care hospital between April 2018 and March 2019, were examined.

The participating hospital was a 900-bed academic hospital. Patients who independently carried out basic activities of daily living (ADL) prior to hospitalization were included in this study. To avoid unequal opportunities to engage in activities during hospital stays, attending nurses routinely obtained ADL information to assess activity during routine clinical practice in the hospital. Additionally, data on all the study variables were collected from the medical records collated during routine medical practice in a hospital context within the Japanese health insurance system. Patients with missing data on body height or weight were excluded from the analyses. Follow-up was conducted from admission until discharge from the hospital. This study was approved by the hospital’s ethics committee (ID: 2019-012). Due to the nature of the retrospective design, the ethics committee waived informed consent. Instead, an opt-out procedure was used to provide all patients with the opportunity to withdraw from participation in the study. The opt-out procedure entailed placing an announcement on the hospital’s web page, so that participants could withdraw from the research at will.

### 2.2. Fall Detection

In the hospital, medical staff were supposed to report unfavorable incidents to the Medical Safety Committee, including, for example, a fall during hospitalization. The reporting system, entailing the collection of every incident related to medical and care practices in a given hospital, including falls, is hospital-determined and based on the Japanese health insurance system, with regular external audits. Medical staff are educated on and encouraged to capture any unfavorable incidents on the system. Incident information is recorded using database software forming part of the electronic medical record system installed in the hospital. In this study, we reviewed evidence of falls based on the data recorded in these reports.

### 2.3. Baseline Parameters

Disease information was classified using the International Classification of Disease-10 (ICD-10) codes, recorded by attending physicians on the medical chart. Diseases that led to hospitalization and comorbidities were extracted from the medical chart. In addition, comorbidities were scored using the Charlson Comorbidity Index (CCI), a predictor of mortality risk [18].

### 2.4. Diagnosis of Malnutrition

Diagnosis of malnutrition was based on the European Society for Clinical Nutrition and Metabolism (ESPEN) definition criteria [19]. As recommended by the Japanese health insurance system, all newly admitted patients were screened for malnutrition at admission. The hospital employed the Mini Nutritional Assessment Short-Form (MNA-SF) as a nutritional screening tool for older adult patients. Furthermore, the system necessitates nutritional assessment so as to enable the provision of hospitalized medical treatment. Information on medical care strategies, including nutritional care at admission, is supposed to be provided on a certain document. If the document is not given to the patient during hospitalization, then the Japanese health insurance system does not cover the full medical costs incurred in hospital. The ESPEN published a new consensus statement on the diagnosis of malnutrition in March 2015 [19]. ESPEN-defined malnutrition is diagnosed in two steps. First, a validated risk screening tool is suggested to identify individuals at risk of malnutrition. In the study, we used the MNA-SF for initial screening, which was administered by trained nurses at admission to the hospital. The MNA-SF is an ordinal scale with a score range of 0 to 14; a score of 11 or less points to a risk of malnutrition [20]. The secondary step defines patients at risk of malnutrition as malnourished, depending on the applicability of one of the following three options to them: 1) BMI <18.5 kg/m^2^, 2) unintentional weight loss >5% over the last 3–6 months, combined with BMI <20 kg/m^2^ if aged <70 years or <22.0  kg/m^2^ if aged ≥70 years, and 3) unintentional weight loss combined with fat-free mass index <15.0 kg/m^2^ if female or <17.0 kg/m^2^ if male. The fat-free mass index (FFMI) is calculated by dividing a patient’s estimated fat-free mass by patient height in meters squared (m^2^). Fat-free mass is obtained using an estimated 24-h urine creatinine excretion rate (eCER) and is calculated as follows: fat-free mass = 13.0 + 0.03 × eCER; eCER (mg/day) = 879.89 + 12.51 × weight (kg) − 6.19 × age (−379.42 if a woman) [21,22]. At hospitalization, all patients were assessed for malnutrition using the relevant information.

### 2.5. Sample Size Calculation

Previous studies have reported that 28.9% of elderly patients admitted to our hospital are malnourished [23]. In addition, approximately 1% of hospitalized older patients had been reported to fall in the hospital, based on the hospital’s unpublished preliminary data. Using these data, the study sample size was calculated in advance. The odds ratio of potential risk factors for falls were reported to be around two [1]; it was assumed that the malnourished group would fall twice as much as the non-malnourished group under the conditions of power = 0.8 and α error = 0.05. Moreover, the study required more than 5782 participants. Therefore, considering the number of older inpatients at the hospital (around 6000 patients per year), the study reviewed all older adult inpatients admitted over a one-year period.

### 2.6. Statistical Analyses

For quantitative variables, parametric variables assessed through a histogram were shown as mean ± standard deviation, and comparisons between groups were analyzed using a t-test. Nonparametric variables were shown as the median (interquartile range) and analyzed using the Mann–Whitney U test. Categorical variables were expressed as frequencies (percentages) and analyzed using a Chi-squared test. A Kaplan–Meier curve analysis was performed to clarify the impact of malnutrition on time and the first incident occurrence, and differences between the groups were examined using a log-rank test. A multivariate Cox regression analysis was performed using covariates which could be confounding factors for subjects’ falls in univariate Cox regression models (*p* < 0.1). The length of the hospital stay at the first fall was adjusted in the Cox regression models. Analyses were performed using SPSS 24.0 software (IBM Japan, Tokyo, Japan) and *p*-values < 0.05 were considered significant.

## 3. Results

**Figure 1:**
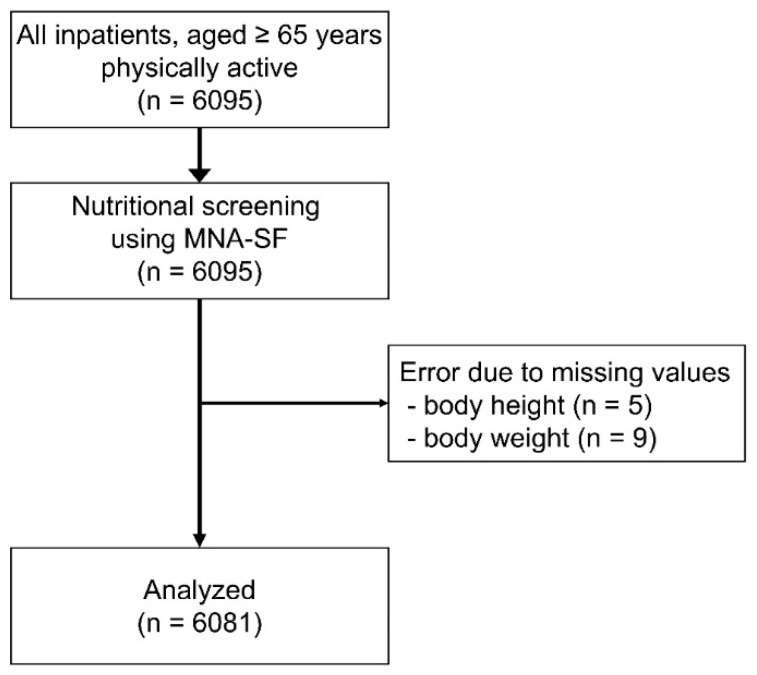
Flowchart of the study. Physically active and consecutive older adult patients (*n* = 6095) were examined for participation eligibility. Eventually, 6081 patients were included in the study. Abbreviation: MNA-SF, Mini Nutritional Assessment Short Form.

During the study period, 6095 elderly patients whose basic ADL was independent prior to the onset of the disease resulting in hospitalization were admitted, and all of them were enrolled and examined to determine eligibility for analysis. Five and nine patients with missing data on height and body weight, respectively, were excluded from the analyses. A total of 6081 patients were included in the final analysis (Figure 1).

All subjects were screened for malnutrition risk at admission using the MNA-SF.

Table 1 shows the characteristics of patients whose data were analyzed. The mean patient age was 74.4 ± 6.1 years and 3535 (58.1%) of patients were men. A total of 1550 patients were at risk of malnutrition based on their MNA-SF scores of ≤11 points; 668 (12.3%) patients were malnourished. Based on the ICD-10, the primary causes of admission were neoplasms (31.1%), diseases of the eye and adnexa (17.2%), diseases of the digestive system (16.1%), diseases of the circulatory system (14.6%), and diseases of the genitourinary system (3.7%).

**Figure 2:**
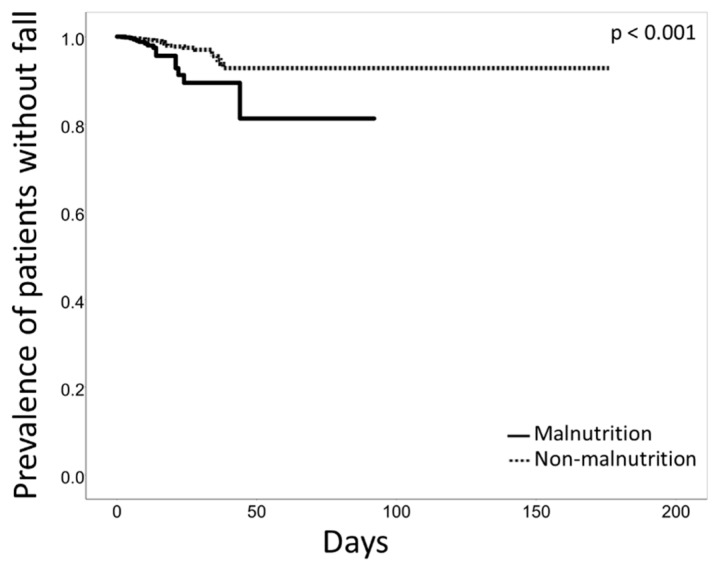
Kaplan–Meier curve analysis for the first fall and the length of the hospital stay. Malnourished patients (solid line) had a higher fall rate than patients without malnutrition (dotted line, log-rank test *p* < 0.001). The number of patients remaining at days 5, 10, 15, 20, 30, and 50 were 449, 263, 138, 77, 26, and 5, respectively, in the malnutrition group; and 2991, 1655, 865, 494, 195, and 46, respectively, in the non-malnutrition group.

In this cohort, 55 patients (0.9%) fell. The malnourished group had a higher fall rate than the non-malnourished group (2.4% vs. 0.7%, *p* < 0.001; Table 2, Figure 2). The median and interquartile range of the length of the hospital stay was 8 (3–13) days for the malnourished group and 5 (2–11) days for the non-malnourished group (*p* < 0.001).

Table 3 shows univariate and multivariate analyses for a fall during hospitalization.

**Figure 3:**
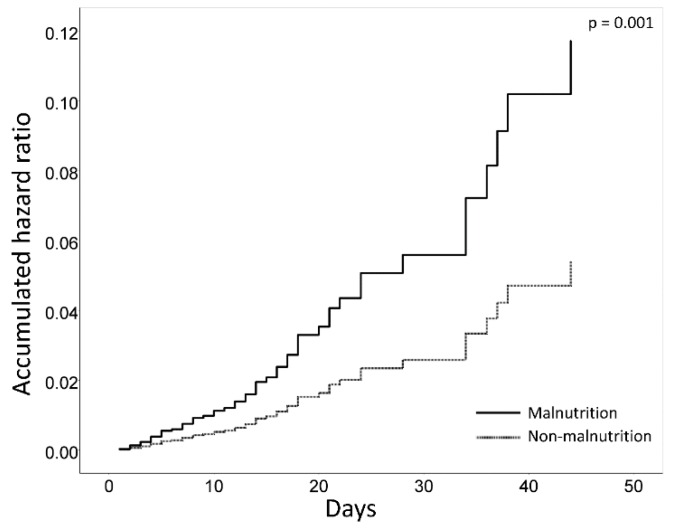
Accumulated hazard ratio over hospitalized days. The multivariate Cox regression model depicted a higher hazard ratio in malnourished patients (solid line) than in patients without malnutrition (dotted line, *p* = 0.001).

In the univariate analysis, age (hazard ratio (HR) = 1.05, 95% CI: 1.01–1.09), sex (male) (HR = 2.53, 95% CI: 1.33–4.80), CCI (HR = 1.11, 95% CI: 1.03–1.20), neoplasms (HR = 0.33, 95% CI: 0.10–1.12), diseases of the musculoskeletal system and connective tissue (HR = 0.10, 95% CI: 0.01–0.94), and malnutrition (HR = 2.67, 95% CI: 1.49–4.77) had a higher hazard ratio for falls. At 1.21 (95% CI 0.70–2.08, *p* = 0.490), the HR of the risk of malnutrition (MNA-SF: 0–11) did not reach statistical significance. Age, sex, CCI, neoplasm, and musculoskeletal system diseases were selected as covariates for multivariate analysis in this study. In multivariate analysis, malnutrition (HR = 2.78, 95% CI: 1.51–5.00, *p* = 0.001) had the highest hazard ratio for falls among covariates. In addition, age (HR = 1.05, 95% CI: 1.01–1.09), sex (male) (HR = 2.50, 95% CI: 1.31–4.79), and CCI (HR = 1.14, 95% CI: 1.04–1.25) were predictors of falls during hospitalization (Figure 3).

## 4. Discussion

In this study, we examined whether the presence of malnutrition was associated with the occurrence of in-hospital falls in elderly patients in acute care hospitals. The risk of in-hospital falls among patients with malnutrition was 2.7 times higher than that among patients without malnutrition. To our knowledge, this is the first study to report an association between malnutrition at admission and falls in an acute care hospital. Implementation of nutritional screening and assessment at admission to hospital would help in the prediction of patients at risk of falls during their hospital stays. The fact that the frequency of falls is reported to be less than 1% among patients admitted to acute care hospitals [24] warrants a large sample size. It may be possible and prove significant to use nutritional status at the time of admission to an acute care hospital to predict in-hospital falls.

Malnutrition had a hazard ratio of 2.7 compared to non-malnutrition; however, malnutrition risk did not reach significance. In this study, we compared the fall rate with and without malnutrition in older in-patients with independent ADL. Previous studies have reported that malnutrition is a risk factor for falls among older adults with independent ADL. Trevisan et al. [17] reported in a systematic review that malnutrition increased fall rates by about 1.5 times among community-dwelling older adults. In addition, the Taiwan Longitudinal Survey on Aging, a national survey of non-institutionalized citizens in Taiwan, found that among the 97.5% of people who can move around, malnutrition risk was associated with falls [25]. However, only one study has reported associations between malnutrition and in-hospital falls in acute hospital settings. In a study in an acute hospital, Lackoff et al. reported that malnutrition (determined by a combination of decreased BMI and Subjective Global Assessment) was not associated with increased in-hospital falls in older patients, whereas an association with harmful falls was detected [26]. That study diagnosed malnutrition on audit day, rather than on admission day, and did not consider the time frame of fall incidences. Furthermore, the hospital in that study specialized in cardiothoracic medicine. The timing of diagnoses, length of hospital stay, and comorbidities might have influenced Lackoff et al.’s study results. Eglseer et al. reported that in a multivariate analysis, in-hospital falls in older patients were not significantly associated with malnutrition [27]. However, their study examined in-hospital falls before assessing malnutrition. A cohort study design might be suitable, as opposed to a case-control design, to detect malnutrition’s prediction of in-hospital falls. Delirium is one of the factors that increases the risk of falls [28]. Acute hospitalization may result in acute changes in mental status (e.g., delirium) [29] and deconditioning of the whole body [30]. Furthermore, many older hospital patients may be likely to be forced to take bed rest in the early phase of hospitalization, despite being able to walk independently [8]. Physical inactivity greatly affects muscle strength and muscle mass [31]. Besides, patients who are malnourished at the time of admission often already have low muscle strength and low muscle mass [32]. Therefore, malnutrition at the time of hospitalization can predispose one to a worsened and easily deteriorating physical condition during hospitalization. In fact, hospital admissions have been reported to reduce muscle mass and strength [33,34].

In this study, there was an association between falls and being older and being male during admission to the acute care hospital. Some study findings are compatible with ours regarding older patients. Plati et al. [35] reported that older hospitalized patients were more likely to fall compared to younger patients based on a case-control study. Church et al. reported an association between being an elderly patient hospitalized for more than 23 h after surgery, and an increased fall rate [36]. Results relating to gender have proved inconsistent. In their study on community-dwelling older adults, Zhao et al. [37] reported that men were more likely to fall than women, as in our results, but another study reported that women were more likely to fall than men mainly in community-dwelling setting [38]. Further research is needed to investigate the association between falls and gender in hospitals. While our results suggested that being older and being male were independently associated with in-hospital falls after adjusting for malnutrition, further research is needed to identify any interaction between these factors and malnutrition in different settings.

The current study found that weighted and accumulated comorbidities were associated with incidences of falls in patients admitted to an acute care hospital. Shumway-Cook et al. reported no differences in the number of comorbidities between people who fell and those who did not [39]. However, these authors reported only the number of comorbidities, but not a weighted index according to the types of comorbidities. The CCI score, as employed in the current study, is known as a comorbidity index with accumulation of weighted comorbidity, which is related to mortality in older adults [18]. Fetal diseases might have an impact on falls during hospitalization. Additionally, it has been reported that patients with a high CCI score were prescribed more drugs than those with a low CCI score during/prior to their admission to acute care hospitals [9]. Polypharmacy, which is known as a risk factor for falls, might also have an impact on falls during hospitalization [40].

This study has some limitations. First, the research was limited to those displaying ADL independence. Therefore, the study results may not be generalizable to all hospitalized older adults. However, since the differences in ADL dependence serve as a confounding factor related to both malnutrition and falls, the study included only one stratification of ADL to eliminate the influence of ADL. Second, the study did not obtain additional participant information such as cognitive and drug-related statuses. Although both these conditions are known to be associated with falls among older adults [41], this information was not recorded in the hospital’s medical recording system, thereby preventing us from linking these conditions with the rest of the collected data. Third, BMI values that determine malnutrition may differ between Europeans and the Japanese [12]. Although many studies have reported ESPEN-defined malnutrition to be associated with malnutrition among Asian populations, a malnutrition diagnosis valid for the population under study would be better.

## 5. Conclusions

Malnutrition at admission to hospital could be a risk factor for in-hospital falls. Falls during hospitalization might be predictable in advance, based on early nutrition screening and the detection of malnutrition at the time of hospitalization in the acute phase. Additionally, adequate nutritional interventions might help in the prevention of falls. Future studies could examine the applicability of these findings across various ADL groups, and the effect of nutritional interventions on falls.

## Figures and Tables

**Table 1:** Characteristics of subjects whose data were analyzed.

	Overall (*n* = 6081)	Malnutrition (*n* = 668)	Non-Malnutrition (*n* = 5413)	*p*-Value
Age, year	74.4 ± 6.1	75.3 ± 6.4	74.3 ± 6.0	<0.001 ^a^
Sex, *n* (%)				
Male	3535 (58.1)	296 (44.3)	3239 (59.8)	<0.001 ^b^
Female	2546 (41.9)	372 (55.7)	2174 (40.2)	
Height (cm)				
Male	165.1 ± 6.0	164.7 ± 6.1	165.2 ± 6.0	0.165 ^a^
Female	151.8 ± 5.7	152.5 ± 6.0	151.7 ± 5.7	0.008 ^a^
Weight (kg)				
Male	62.5 ± 10.0	47.9 ± 5.8	63.8 ± 9.2	<0.001 ^a^
Female	51.8 ± 9.2	40.8 ± 4.7	53.6 ± 8.4	<0.001 ^a^
Body mass index (kg/m^2^)				
Male	22.9 ± 3.2	17.7 ± 1.7	23.4 ± 2.8	<0.001 ^a^
Female	22.5 ± 3.6	17.5 ± 1.6	23.3 ± 3.2	<0.001 ^a^
FFMI (kg/m^2^)				
Male	18.0 ± 1.4	16.0 ± 1.0	18.2 ± 1.3	<0.001 ^a^
Female	14.6 ± 1.5	12.7 ± 0.8	14.9 ± 1.4	<0.001 ^a^
Charlson Comorbidity Index, score	2.3 ± 2.8	3.0 ± 3.2	2.2 ± 2.7	<0.001 ^a^
MNA-SF, score	13 (11–14)	11 (9–11)	13 (12–14)	<0.001 ^c^
12–14, *n* (%)	4,531 (74.5)	0 (0.0)	4,531 (83.7)	<0.001 ^b^
8–11, *n* (%)	1,483 (24.4)	620 (92.8)	863 (15.9)	
0–7, *n* (%)	67 (1.1)	48 (7.2)	19 (0.4)	
Any recent weight loss, *n* (%)	808 (13.3)	223 (33.4)	585 (10.8)	<0.001 ^b^
Weight loss > 5%, *n* (%)	205 (3.4)	145 (21.7)	60 (1.1)	<0.001 ^b^

Patients in the malnutrition and non-malnutrition groups were compared on each characteristic. The symbols, ^a^, ^b^, and ^c^ represent the *t*-test, the Chi-squared test, and the Mann–Whitney U test employed for comparisons. Abbreviation: FFMI, fat-free mass index; MNA-SF, Mini Nutritional Assessment Short Form.

**Table 2:** Outcomes during hospitalization.

	Overall (*n* = 6208)	Malnutrition (*n* = 682)	Non-Malnutrition (*n* = 5526)	*p*-Value
Incidence of a fall				
Yes (%)	55 (0.9)	16 (2.4)	39 (0.7)	<0.001
No (%)	6026 (99.1)	652 (97.6)	5374 (99.3)	

Patients in the malnutrition and non-malnutrition groups were compared. The Chi-squared test was employed for comparison.

**Table 3:** Univariate and multivariate analysis for in-hospital falls.

	Univariate			Multivariate		
	HR	95% CI	*p*-Value	HR	95% CI	*p*-Value
Age	1.05	1.01–1.09	0.013	1.05	1.01–1.09	0.022
Sex (male)	2.53	1.33–4.80	<0.001	2.50	1.31–4.79	0.006
Charlson Comorbidity Index *	1.11	1.03–1.20	0.005	1.14	1.04–1.25	0.004
Disease						
Neoplasms	0.33	0.10–1.12	0.075	0.61	0.34–1.10	0.101
Diseases of the eye and adnexa	0.16	0.02–1.64	0.124			
Diseases of the digestive system	0.38	0.09–1.64	0.194			
Diseases of the circulatory system	0.49	0.14–1.74	0.270			
Diseases of the genitourinary system	0.44	0.07–2.71	0.379			
Diseases of the nervous system	0.00	0.00–Inf.	0.980			
Diseases of the respiratory system	0.56	0.13–2.53	0.453			
Diseases of the musculoskeletal system and connective tissue	0.10	0.01–0.94	0.044	0.40	0.05–2.97	0.371
Injury, poisoning, and certain other consequences of external causes	0.00	0.00–Inf.	0.968			
Endocrine, nutritional, and metabolic diseases	0.30	0.05–1.83	0.192			
Diseases of the ear and mastoid process	0.60	0.06–5.86	0.659			
Certain infectious and parasitic diseases	1.00	reference	0.758			
Diseases of the blood and blood-forming organs and certain disorders involving the immune mechanism	0.44	0.05–4.22	0.475			
Diseases of the skin and subcutaneous tissue	1.31	0.14–12.70	0.814			
Mental and behavioral disorders	1.07	0.22–5.32	0.934			
Others	0.00	0.00–Inf.	0.990			
Malnutrition	2.67	1.49–4.77	<0.001	2.78	1.51–5.00	0.001

Abbreviations: HR, hazard ratio; CI, confidence interval; Inf., infinity. * When performing multivariate analysis, cancer patients subtracted malignancy (2 points) from the Charlson Comorbidity Index score.

## References

[1] Deandrea S., Lucenteforte E., Bravi F., Foschi R., La Vecchia C., Negri E. (2010). Risk factors for falls in community-dwelling older people: A systematic review and meta-analysis. Epidemiology.

[2] Pfortmueller C.A., Lindner G., Exadaktylos A.K. (2014). Reducing fall risk in the elderly: Risk factors and fall prevention, a systematic review. Minerva Med..

[3] Hendrich A., Nyhuis A., Kippenbrock T., Soja M.E. (1995). Hospital falls: Development of a predictive model for clinical practice. Appl. Nurs. Res..

[4] Ambrose A.F., Paul G., Hausdorff J.M. (2013). Risk factors for falls among older adults: A review of the literature. Maturitas.

[5] Rubenstein L.Z., Josephson K.R. (2002). The epidemiology of falls and syncope. Clin. Geriatr Med..

[6] Tanaka S., Kamiya K., Hamazaki N., Matsuzawa R., Nozaki K., Maekawa E., Noda C., Yamaoka-Tojo M., Matsunaga A., Masuda T. (2017). Utility of SARC-F for Assessing Physical Function in Elderly Patients With Cardiovascular Disease. J. Am. Med. Dir. Assoc..

[7] Chen L.K., Woo J., Assantachai P., Auyeung T.W., Chou M.Y., Iijima K., Jang H.C., Kang L., Kim M., Kim S. (2020). Working Group for Sarcopenia: 2019 Consensus Update on Sarcopenia Diagnosis and Treatment. J. Am. Med. Dir. Assoc..

[8] Brown C.J., Redden D.T., Flood K.L., Allman R.M. (2009). The underrecognized epidemic of low mobility during hospitalization of older adults. J. Am. Geriatr Soc..

[9] Frenkel W.J., Jongerius E.J., Mandjes-van Uitert M.J., van Munster B.C., de Rooij S.E. (2014). Validation of the Charlson Comorbidity Index in acutely hospitalized elderly adults: A prospective cohort study. J. Am. Geriatr Soc..

[10] Lim S.L., Ong K.C., Chan Y.H., Loke W.C., Ferguson M., Daniels L. (2012). Malnutrition and its impact on cost of hospitalization, length of stay, readmission and 3-year mortality. Clin. Nutr..

[11] Maeda K., Koga T., Akagi J. (2018). Nutritional variables predict chances of returning home and activities of daily living in post-acute geriatric care. Clin. Interv. Aging.

[12] Maeda K., Ishida Y., Nonogaki T., Mori N. (2020). Reference body mass index values and the prevalence of malnutrition according to the Global Leadership Initiative on Malnutrition criteria. Clin. Nutr..

[13] Jensen G.L., Wheeler D. (2012). A new approach to defining and diagnosing malnutrition in adult critical illness. Curr. Opin. Crit. Care.

[14] Sullivan D.H., Sun S., Walls R.C. (1999). Protein-energy undernutrition among elderly hospitalized patients: A prospective study. JAMA.

[15] Moreland J.D., Richardson J.A., Goldsmith C.H., Clase C.M. (2004). Muscle weakness and falls in older adults: A systematic review and meta-analysis. J. Am. Geriatr. Soc..

[16] Reijnierse E.M., Verlaan S., Pham V.K., Lim W.K., Meskers C.G.M., Maier A.B. (2019). Lower Skeletal Muscle Mass at Admission Independently Predicts Falls and Mortality 3 Months Post-discharge in Hospitalized Older Patients. J. Gerontol. A Biol. Sci. Med. Sci..

[17] Trevisan C., Crippa A., Ek S., Welmer A.K., Sergi G., Maggi S., Manzato E., Bea J.W., Cauley J.A., Decullier E. (2019). Nutritional Status, Body Mass Index, and the Risk of Falls in Community-Dwelling Older Adults: A Systematic Review and Meta-Analysis. J. Am. Med. Dir. Assoc..

[18] Charlson M.E., Pompei P., Ales K.L., MacKenzie C.R. (1987). A new method of classifying prognostic comorbidity in longitudinal studies: Development and validation. J. Chronic Dis..

[19] Cederholm T., Bosaeus I., Barazzoni R., Bauer J., Van Gossum A., Klek S., Muscaritoli M., Nyulasi I., Ockenga J., Schneider S.M. (2015). Diagnostic criteria for malnutrition—An ESPEN Consensus Statement. Clin. Nutr..

[20] Rubenstein L.Z., Harker J.O., Salva A., Guigoz Y., Vellas B. (2001). Screening for undernutrition in geriatric practice: Developing the short-form mini-nutritional assessment (MNA-SF). J. Gerontol. A Biol. Sci. Med. Sci..

[21] Ix J.H., Wassel C.L., Stevens L.A., Beck G.J., Froissart M., Navis G., Rodby R., Torres V.E., Zhang Y.L., Greene T. (2011). Equations to estimate creatinine excretion rate: The CKD epidemiology collaboration. Clin. J. Am. Soc. Nephrol..

[22] Jassal S.K., Wassel C.L., Laughlin G.A., Barrett-Connor E., Rifkin D.E., Ix J.H. (2015). Urine creatinine-based estimates of fat-free mass in community-dwelling older persons: The Rancho Bernardo study. J. Ren. Nutr..

[23] Ishida Y., Maeda K., Nonogaki T., Shimizu A., Yamanaka Y., Matsuyama R., Kato R., Mori N. (2019). Impact of edema on length of calf circumference in older adults. Geriatr. Gerontol. Int..

[24] Koh S.S., Manias E., Hutchinson A.M., Johnston L. (2007). Fall incidence and fall prevention practices at acute care hospitals in Singapore: A retrospective audit. J. Eval. Clin. Pract..

[25] Tsai A.C., Lai M.Y. (2014). Mini Nutritional Assessment and short-form Mini Nutritional Assessment can predict the future risk of falling in older adults - results of a national cohort study. Clin. Nutr..

[26] Lackoff A.S., Hickling D., Collins P.F., Stevenson K.J., Nowicki T.A., Bell J.J. (2020). The association of malnutrition with falls and harm from falls in hospital inpatients: Findings from a 5-year observational study. J. Clin. Nurs..

[27] Eglseer D., Hoedl M., Schoberer D. (2020). Malnutrition risk and hospital-acquired falls in older adults: A cross-sectional, multicenter study. Geriatr. Gerontol. Int..

[28] Sillner A.Y., Holle C.L., Rudolph J.L. (2019). The Overlap Between Falls and Delirium in Hospitalized Older Adults: A Systematic Review. Clin. Geriatr. Med..

[29] Inouye S.K., Schlesinger M.J., Lydon T.J. (1999). Delirium: A symptom of how hospital care is failing older persons and a window to improve quality of hospital care. Am. J. Med..

[30] Krumholz H.M. (2013). Post-hospital syndrome—An acquired, transient condition of generalized risk. N. Engl. J. Med..

[31] Coker R.H., Wolfe R.R. (2012). Bedrest and sarcopenia. Curr. Opin. Clin. Nutr. Metab. Care.

[32] Sanchez-Rodriguez D., Marco E., Ronquillo-Moreno N., Miralles R., Vazquez-Ibar O., Escalada F., Muniesa J.M. (2017). Prevalence of malnutrition and sarcopenia in a post-acute care geriatric unit: Applying the new ESPEN definition and EWGSOP criteria. Clin. Nutr..

[33] Hartley P., Costello P., Fenner R., Gibbins N., Quinn E., Kuhn I., Keevil V.L., Romero-Ortuno R. (2019). Change in skeletal muscle associated with unplanned hospital admissions in adult patients: A systematic review and meta-analysis. PLoS ONE.

[34] Cruz-Jentoft A.J., Sayer A.A. (2019). Sarcopenia. Lancet.

[35] Plati C., Lanara V., Mantas J. (1992). Risk factors responsible for patients’ falls. Scand. J. Caring Sci..

[36] Church S., Robinson T.N., Angles E.M., Tran Z.V., Wallace J.I. (2011). Postoperative falls in the acute hospital setting: Characteristics, risk factors, and outcomes in males. Am. J. Surg..

[37] Zhao Y.L., Alderden J., Lind B., Stibrany J. (2019). Risk factors for falls in homebound community-dwelling older adults. Public Health Nurs..

[38] Peng K., Tian M., Andersen M., Zhang J., Liu Y., Wang Q., Lindley R., Ivers R. (2019). Incidence, risk factors and economic burden of fall-related injuries in older Chinese people: A systematic review. Inj. Prev..

[39] Shumway-Cook A., Ciol M.A., Hoffman J., Dudgeon B.J., Yorkston K., Chan L. (2009). Falls in the Medicare population: Incidence, associated factors, and impact on health care. Phys. Ther..

[40] Seppala L.J., van de Glind E.M.M., Daams J.G., Ploegmakers K.J., de Vries M., Wermelink A., van der Velde N., Task E., Finish Group on Fall-Risk-Increasing D. (2018). Fall-Risk-Increasing Drugs: A Systematic Review and Meta-analysis: III. Others. J. Am. Med. Dir. Assoc..

[41] Kropelin T.F., Neyens J.C., Halfens R.J., Kempen G.I., Hamers J.P. (2013). Fall determinants in older long-term care residents with dementia: A systematic review. Int. Psychogeriatr..

